# Detection, identification and genotyping of *Borrellia* spp. in rodents in Slovenia by PCR and culture

**DOI:** 10.1186/s12917-015-0501-y

**Published:** 2015-08-08

**Authors:** Tjaša Cerar, Miša Korva, Tatjana Avšič-Županc, Eva Ružić-Sabljić

**Affiliations:** Faculty of Medicine, Institute of Microbiology and Immunology, University of Ljubljana, Zaloška 4, 1000 Ljubljana, Slovenia

**Keywords:** *Borrelia burgdorferi* sensu lato, *B. miyamotoi*, Rodents, Molecular methods, Isolation, *Mlu*I-LRFP, MLST

## Abstract

**Background:**

*Borrelia burgdorferi* sensu lato, the agent of Lyme borreliosis, is mainly maintained in natural foci through the transmission cycles of competent tick vectors (*Ixodes* sp.) and a vertebrate reservoir. Specific rodents have been identified as the principal reservoir of *Borrelia burgdorferi* sensu lato in Europe. *Borrelia miyamotoi* is the only relapsing fever spirochete transmitted by the same tick. The aim of the present study was to perform an epidemiological survey to determine the presence of *B. burgdorferi* sensu lato in rodents occurring in Slovenia and to explore the presence of *Borrelia miyamotoi*.

The study was performed in two parts, retrospective and prospective; a total of 297 rodents was analyzed. Detection and identification of borrelia was performed by molecular methods and additionally in the prospective study by isolation and genotyping (*Mlu*I-LRFP and MLST).

**Results:**

During the prospective part of the study, borrelia was isolated from 2/46 (4.3 %) lung specimens and from 10/46 (21.7 %) heart specimens of rodents. All isolated strains were identified as *B. afzelii* subtype Mla1, and MLST analysis revealed 5 distinct sequence types. Borrelia DNA was successfully detected by one or other of the PCR methods in 18/46 (39.1 %) and 75/251 (29.9 %) samples in the prospective and retrospective studies, respectively. LightMix® was found to be more sensitive than the ‘’in-house” nested PCR (91/297 (30.6 %) vs 48/297 (16.1 %)).

*Borrelia miyamotoi* DNA was detected in 1/251 (0.4 %) and in 1/46 (2.2 %) heart specimens, in the retrospective and prospective parts of the study, respectively.

**Conclusion:**

We determined the prevalence of *B. afzelii* in rodents and report for the first time the presence of *B. miyamotoi* in Slovenia.

## Background

*Borrelia burgdorferi* sensu lato is the causative agent of Lyme borreliosis, the most common tick transmitted infection in Slovenia: in 2012, the incidence of the disease was 239.8 cases/100,000 inhabitants [1]. At least four species are among the most important pathogenic species in Europe, *Borrelia afzelii*, *Borrelia garinii*, *Borrelia burgdorferi* sensu stricto and *Borrelia spielmanii*, forming part of the *B. burgdorferi* sensu lato complex [2]. All strains are mainly maintained in natural foci through the transmission cycles of competent tick vectors and a vertebrate reservoir. Specific rodents have been identified as the principal reservoir host of *B. burgdorferi* sensu lato in Europe, such as wood mouse (*Apodemus sylvaticus*), yellow-necked mouse (*Apodemus flavicollis*) and bank vole (*Clethrionomys glareous*) [3, 4]. Several other vertebrates have also been described as competent reservoir hosts, such as birds, dormice, hedgehogs, rats, squirrels, hares and lizards [5]. Borrelia is maintained for long time periods in a competent reservoir host, often lifelong [5].

Infected *Ixodes* ticks transmit borrelia to mammals through a bite. In Europe and Asia, *Ixodes ricinus* and *Ixodes persulcatus* ticks are the main vectors for *B. garinii*, *B. afzelii*, and *B. burgdorferi* sensu stricto transmission, while the deer tick *Ixodes scapularis* transmits *B. burgdorferi* sensu stricto in North America [6].

*Borrelia miyamotoi* is the only relapsing fever spirochete transmitted by hard ticks of the *Ixodes* species. Infection with *B. miyamotoi* has recently been reported to be associated with symptomatic febrile illnesses in humans in Eurasia and North America [7–9]. *B. miyamotoi* has been detected in urinary bladder and/or blood of wild rodents *Apodemus argenteus*, *Apodemus speciosus*, *Myodes rufocanus* and *Myodes rutilus* [10].

The aim of the present study was to perform an epidemiological survey to determine the presence of *B. burgdorferi* sensu lato in rodents occurring in Slovenia and to explore the presence of *Borrelia miyamotoi*. Additionally, we wanted to compare the results of two specific PCR, amplifying different parts of the *ospA* gene, in order to determine the presence of borrelia in two different rodent tissues: heart and lung.

## Methods

The study was performed in two parts, retrospective and prospective.

In the retrospective part, we analyzed a total of 251 heart biopsies from rodents (105 females, 146 males) live trapped in spring and autumn of 2012 in the Central Slovenian region.

All rodents were identified to species level and heart biopsies were stored at −80 °C until tested.

The prospective part of the study took place in spring and autumn of 2013; 46 rodents (24 females, 23 males) were live trapped in the Central Slovenian region. After identification to species level, lungs and hearts were removed. Each specimen was dissected into two equal parts: one was immediately inoculated into modified Kelly-Pettenkofer medium and incubated at 33 °C for borrelia isolation, [11] while the other was frozen at −80 °C for PCR analysis.

### Identification of rodents

Morphological species determination was confirmed by PCR and sequencing of the partial mitochondrial cytochrome b gene [12]. Rodent species were determined by sequence comparisons using the BLAST algorithm (www.ncbi.nlm.nih.gov).

### Culture, isolation and characterization of borrelia strains

The lung and heart samples from the prospective study were incubated at 33 °C and examined weekly for the presence of spirochetes using dark-field microscopy. The samples were considered negative if no growth was detected after 9 weeks of incubation [13].

Genotypic characterization of isolated borrelia strains was performed using large restriction fragment pattern (LRFP) after *Mlu*I restriction and multilocus sequence typing (MLST) analysis.

*Mlu*I-LRFP was performed as previously described [13]. Briefly, borrelial DNA was isolated by the gel insert method, digested with restriction endonuclease *Mlu*I, and restricted fragments were separated by pulse-field gel electrophoresis (run time: 24 h, ramping time: 3–40 s). *Borrelia* species and subtypes were identified by their specific restriction profiles [13].

For MLST analyses, borrelial DNA was isolated using the InstaGene matrix (BioRad, USA) according to the manufacturer’s instructions. Eight chromosomal housekeeping genes were amplified by nested PCR and sequenced in both directions, as previously described by Margos et al. [14]. Sequences were analyzed using CLC Main Workbench 6.9.1, MLST Module (CLC bio, Denmark) and assigned allelic numbers and sequence types. New sequence types were added to the MLST database hosted by Imperial College London (London, UK) and are accessible at the MLST website (http://borrelia.mlst.net/).

### Nucleic acid isolation from tissue specimens

DNA was isolated from heart and lung biopsies according to the manufacturer’s recommendations using a MagNA Pure Compact Total Nucleic Acid Isolation Kit, Roche, Germany and QIAamp DNA Mini Kit, Qiagen, in prospective and retrospective parts of the study, respectively.

### Detection of *Borrelia burgdorferi* sensu lato DNA

The presence of *B. burgdorferi* sensu lato DNA was determined using two different PCRs, ‘’in-house” nested PCR and commercial real-time PCR (LightMix® Kit for detection of *Borrelia* spp., TIB MOLBIOL GMBh, Germany), both targeting the *ospA* gene.

### LightMix® Kit for detection of *Borrelia* spp

A fragment from the *Borrelia* ospA gene was amplified with specific primers and detected with hybridization probes according to the manufacturer’s recommendations. In addition to amplification, the kit enables identification of *Borrelia* species by melting temperature (Tm) analysis. The PCR reaction is monitored by an additional PCR product to detect possible inhibition.

### In-house nested PCR of ospA

Nested PCR was performed using primers for amplification of the *ospA* gene described by Guy and Stanek [15], under the following conditions. The reaction was carried out in 30 cycles of 95 °C for 45 s, 50 °C for 45 s and 72 °C for 60 s. Each sample was transferred to a second reaction and amplified under the same conditions for another 30 cycles [16]. One positive and three negative control samples were included in each experiment to control for amplification and contamination. To avoid PCR contamination and amplicon carry-over, samples were processed in separate rooms, and the use of plugged pipette tips was obligatory [17]. PCR amplification resulted in a 351 bp product. Amplification products were analyzed on SYBR Safe DNA gel stain (Invitrogen, USA) stained 1 % agarose gels. Identification of the species was done by sequencing the PCR product. Amplicons were purified and sequenced on ABI3500 (Applied Biosystem, California, USA). Sequences were analyzed with CLC Main Workbench 6.0 (CLC Bio, Denmark) and compared with the BLAST database (www.ncbi.nlm.nih.gov/blast/Blast.cgi).

### Detection of *Borrelia miyamotoi*

The presence of *B. miyamotoi* DNA was detected using a real-time protocol and specific primers targeting the 16S rRNA gene, as described by Platonov et al. [8]. To confirm the specificity of the real-time protocol, an amplified flagellin gene was direct sequenced into all positive samples [8]. Nucleotide sequences were aligned, compared and analyzed by CLC Main Workbench 6.0 (CLC Bio, Denmark) and BLAST (www.ncbi.nlm.nih.gov/blast/Blast.cgi).

### Statistical analysis

Statistical analyses were performed using SPSS Statistics 18.0 (Chicago: SPSS Inc). A 95 % confidence interval (CI) was calculated for the prevalence of *Borrelia* sp. in rodents.

### Ethical statement

Rodents used in this study were collected from nature in collaboration with the Slovenian Museum of Natural History, with the approval of the Ministry of Agriculture, Forestry and Food, and the Administration of the Republic of Slovenia for Food Safety, Veterinary Sector and Plant Protection (323-02-251/2004/7).

## Results

### Identification of rodents

The study included 297 rodent species, 46 in the prospective and 251 in the retrospective part.

During the prospective study, eighteen out of 46 (39.1 %) rodents were identified as yellow necked mice (*Apodemus flavicollis*), 22/46 (47.8 %) as bank voles (*Myodes glareolus*), 5/46 (10.9 %) as Mediterranean water shrews (*Neomys anomalus*) and 1/46 (2.2 %) as a lesser white-toothed shrew (*Crocidura suaveolens*).

The retrospective study included 251 rodents, of which 155 (61.8 %) were identified as yellow necked mice (*Apodemus flavicollis*), 94 (37.5 %) as bank voles (*Myodes glareolus*), and 2 (0.8 %) as wood mice (*Apodemus sylvaticus*).

### Cultivation, isolation and genotypic characterization of borrelia strains

Cultivation was performed on 46 rodents collected in the prospective part of the study. *Borrelia* sp. was isolated from 2/46 (4.3 %) lung specimens and from 10/46 (21.7 %) heart specimens of 7 *Myodes glareolus* and 4 *Apodemus flavicollis. Borrelia* sp. was simultaneously isolated from heart and lung specimens of 1 *Myodes glareolus* (Table 1).

**Table 1:** Genotypic characterization employing *Mlu*I-RFLP and MLST of isolated *Borrelia* strains isolated from lung and heart specimens of different rodents

Rodent genus and species	Lung		Heart	
	MluI-RFLP	MLST ST	RFLP	MLST ST
*Apodemus flavicollis*	culture negative		*B. afzelii* Mla1	335
*Apodemus flavicollis*	culture negative		*B. afzelii* Mla1	335
*Apodemus flavicollis*	culture negative		*B. afzelii* Mla1	335
*Apodemus flavicollis*	culture negative		*B. afzelii* Mla1	335
*Myodes glareolus*	*B. afzelii* Mla1	549^a^	*B. afzelii* Mla1	549^a^
*Myodes glareolus*	culture negative		*B. afzelii* Mla1	549^a^
*Myodes glareolus*	culture negative		*B. afzelii* Mla1	342
*Myodes glareolus*	culture negative		*B. afzelii* Mla1	550^a^
*Myodes glareolus*	culture negative		*B. afzelii* Mla1	215
*Myodes glareolus*	*B. afzelii* Mla1	335	culture negative	
*Myodes glareolus*	culture negative		*B. afzelii* Mla1	335

^a^new ST

*Mlu*I-LRFP **-** Large restriction fragment pattern after MluI digestion on whole genom

MLST – multilocus sequence typing

ST – sequence type

*Mlu*I-LRFP analysis revealed all isolates as *B. afzelii* Mla1. Results are shown in Table 1.

MLST analysis of 12 isolated *B. afzelii* strains revealed 5 distinct sequence types (ST); 3 have been previously reported, 2 were assigned a new ST number (Table 1).

### Detection of borrelial DNA

#### Prospective study

In lung specimens, borrelial DNA was detected in 10/46 (21.7 %) and 8/46 (17.4 %) rodents, using LightMix® and ‘’in-house” nested PCR, respectively; the concordance of the two PCR methods was 42/46 (91.3 %) (Table 2). In heart specimens, borrelial DNA was detected in 17/46 (37.0 %) and 18/46 (39.1 %) rodents with LightMix® and ‘’in-house” nested PCR, respectively; the concordance of the two tests was 45/46 (97.8 %). *Borrelia* sp. DNA was detected by one or other of the applied methods in 18/46 (39.1 %, 95 % CI: 25.0–53.2 %) rodent samples.

**Table 2:** Detection of *Borrelia* sp DNA in lungs and heart of rodents using LightMix® and ‘’in house” nested PCR, both targeting *ospA* gene

Findings	Sample	*Apodemus flavicollis*	*Myodes glareolus*	*Apodemus sylvaticus*	*Apodemus flavicollis*	*Myodes glareolus*	*Neomys anomalus*	*Crocidura suaveolens*
		*Retrospective (year 2012)*			*Prospective (year 2013)*			
*ospA* pos	*Lungs*	ND			1	6	0	0
*LightMix* pos	*heart*	13	17	2	9	8	0	0
*ospA* neg	*Lungs*	ND			13	15	5	1
*LightMix* neg	*heart*	114	63	0	9	13	5	1
*ospA* neg	*Lungs*	ND			3	0	0	0
*LightMix* pos	*heart*	28	14	0	0	0	0	0
*ospA* pos	*Lungs*	ND			0	1	0	0
*LightMix* neg	*heart*	0	0	0	0	1	0	0
All	*Lungs*	ND			18	22	5	1
	*heart*	155	94	2	18	22	5	1

ND – not done

Identification according to Tm using LightMix® revealed the presence of *B. afzelii*/*B. valaisiana*; LightMix® kit does not allow a distinction between these two species based on differences in melting temperatures [18]. Sequencing of ‘’in-house” OspA PCR products revealed the presence of *B. afzelii*, only.

### Retrospective study

Borrelial DNA was detected in 74/251 (29.5 %) and 30/251 (12.0 %) heart biopsies with LightMix® and ‘’in-house” nested PCR, respectively; the concordance of the two tests was 209/251 (83.3 %) (Table 2). *Borrelia* sp. DNA was detected by one or other of the applied methods in 75/251 (29.9 %, 95%CI: 24.2–35.6 %) rodent samples.

As previously reported, identification using Tm analysis revealed the presence of *B. afzelii*/*B. valaisiana;* sequencing of ‘’in-house” OspA PCR products revealed the presence of *B. afzelii*, only.

### Detection of *Borrelia miyamotoi*

*Borrelia miyamotoi* DNA was detected in 1/251 (0.4 %) heart specimens of *Apodemus flavicollis* in the retrospective part of the study and in 1/46 (2.2 %) heart specimens of *Apodemus flavicollis* in the prospective part of the study.

Direct sequencing of PCR product of the flagellin gene of both positive samples revealed 99.58 % and 99.33 % identity with *B. miyamotoi* strain KT12F-IR and *B. miyamotoi* strain OS179m-07, respectively.

## Discussion

*Borrelia burgdorferi* sensu lato utilizes an extremely wide range of hosts, including rodents, birds and lagomorphs [19]. Birds are mainly host for *B. garinii*, whereas *B. afzelii*, *B. burgdorferi* sensu stricto and *B. bavariensis* utilize rodents as their main reservoir host [20]. In Europe, *Apodemus* spp. and *M. glareolus* have been reported to transmit *B. miyamotoi* to 23.8 % of xenodiagnostic ticks [21].

Since the study by Zore et al. [22], no epidemiological data concerning the presence *B. burgdorferi* sensu lato in rodents and ticks have been reported from Slovenia. In addition, the presence of *Borrelia miyamotoi* in Slovenia was neither screened nor previously reported.

In the prospective part of the study, detection of borrelia DNA was successful in 18/46 (39.1 %) samples, while the corresponding result for the retrospective part of the study was 75/251 (29.9 %), detected by one or other of the applied methods. Comparing the two PCR methods, LightMix® was found to be more sensitive than the ‘’in-house” nested PCR (91/297 (30.6 %) vs 48/297 (16.1 %)). Benefits of LightMix® also include ease of performance, rapidity, ability of species discrimination and lower chance of contamination.

The prevalence of borrrelia infection in host animals in our study is comparable with that reported in the study by Zore et al. [22], in which borrelial DNA was detected in 17/34 rodents (50 % prevalence). The prevalence reported in our study is higher than in the study by Schmidt et al. [23], in which borrelia DNA was detected in 14.8 % (16/110) of rodents trapped in the northern part of Austria. Generally, the reasons for differences in prevalence among different studies can be explained by different methods of sample collection, sample sizes, detection methods, geographic locations and time periods [24]. A reliable and standardized approach for such studies is not defined.

Identification according to Tm analysis of the *ospA* gene using LightMix® revealed the presence of *B. afzelii* or *B. valaisiana*; such an analysis does not allow a distinction between these two species, which is a great disadvantage of the kit [18]. Since LightMix is a commercial real-time PCR and sequences of the primers and hybridization probes are not available, sequencing of the product is not possible, which is the main limitation of the assay. On the other hand, comparison of the sequence of our in-house PCR, targeting the *ospA* gene, with the NCBI Blast database revealed the presence of *B. afzelii*, only. These results are in accordance with the study by Schmidt et al., in which *B. afzelii* was identified in the majority of the rodents skin samples [23]. In the study by Perez et al., [25] *B. afzelii* was also the dominant species and the authors concluded that rodents transmitted only *B. afzelii* to ticks. In the previously performed Slovenian study, the most commonly found species was *B. afzelii*, but *B. burgdorferi* sensu stricto and *B. garinii* were also detected [22].

We were able to isolate borrelia from heart and/or lung of 4/18 (22.2 %) yellow-necked mice (*Apodemus flavicollis*), 6/22 (27.3 %) bank voles (*Myodes glareolus*) and from none of the Mediterranean water shrews (*Neomys anomalus*) or lesser white-toothed shrew (*Crocidura suaveolens*). All isolated strains were identified using *Mlu*I-LRFP as *B. afzelii* subtype Mla1. Although we expected *B. garini* and *B. burgdorferi* sensu stricto to be isolated in our study, already reported in Slovenian rodents [22], we did not find them.

Our results are consistent with the results of previous studies, in which it was shown that rodent sera is borreliacidal for *B. garinii* [26]. It was additionally found that the wood mouse is far more competent for *B. afzelii* than for *B. burgdorferi* sensu stricto [27] and that *B. afzelii* is specifically maintained by European rodents [28].

Borrelia sp. can be isolated and/or PCR detected in various organs of small mammals [29]. In the study by Khanakah et al., it was demonstrated that *Borrelia* strains were more frequently cultured from specimens of the bladder wall than from heart muscle. On the other hand, PCR of heart specimens was more often positive than culture of bladder wall and heart muscle [30]. To our knowledge, no study comparing PCR detection in heart, lung and urinary bladder has been published. The main limitation of our study is the absence of urinary bladder samples.

MLST analysis of 12 isolates from rodent specimens revealed 5 distinct sequence types (Table 1); 3 ST have been previously identified, while 2 ST were assigned new ST numbers (Table 1). *B. afzelii* ST 335, which was found in 6 Slovenian isolates, was also previously identified in two Italian isolates from *I. ricinus* ticks, *B. afzelii* ST 215 (found in one Slovenian isolate) in three isolates originating from Latvia and one from Italy, *B. afzelii* ST 342 (found in one Slovenian isolate) in one isolate from *I. ricinus* from Austria (http://borrelia.mlst.net/sql/burstspadvanced.asp).

Both *B. afzelii* isolates with new sequence types (ST549 and 550) originated from *Myodes glareous* and were added to the MLST database.

*Borrelia miyamotoi* DNA was detected in only two heart samples of *A. flavicollis* mice, resulting in a low overall prevalence of 2/297 (0.7 %); more precisely in 0.4 % and 2.2 % in the retrospective and prospective parts of the study, respectively. The results of the study provide the first evidence of *B. miyamotoi* presence in Slovenia.

In the study by Cosson et al. [31], the prevalence of *B. miyamotoi* in ticks and bank voles was 3.0 % (8/267 ticks) and 5.55 % (4/72 bank voles), respectively. A prevalence of 1.5 % was determined in the study by Taylor et al. [10], in which urinary bladders of rodents were tested. In terms of the prevalence of Lyme borreliosis borrelia, *B. miyamotoi* is rarely found in reservoir hosts in Slovenia.

## Conclusion

We determined the prevalence of *B. burgdorferi* sensu lato in various rodents in Slovenia. Using molecular methods, detection of borrelia DNA was successful in 18/46 (39.1 %) and 75/251 (29.9 %) samples, in the prospective and retrospective parts of the study, respectively. LightMix® was found to be more sensitive than ‘’in-house” nested PCR (91/297 (30.6 %) vs 48/297 (16.1 %)), easy to perform, fast and not prone to contamination.

Identification of *Borrelia* species revealed the presence of *B. afzelii*. We report the presence of *B. miyamotoi* for the first time in Slovenia.

## Abbreviations

PCR: Polymerase chain reaction
DNA: Deoxyribonucleic acid
BLAST: Basic local alignment search tool
*ospA*: gen for the major outer surface protein A
LRFP: Large restriction fragment pattern
MLST: Multilocus sequence typing
Tm: Melting temperature
ST: Sequence type
